# MRI Segmentation of Cervical Muscle Volumes in Survived Strangulation: Is There an Association between Side Differences in Muscle Volume and the Handedness of the Perpetrator? A Retrospective Study

**DOI:** 10.3390/diagnostics12030743

**Published:** 2022-03-18

**Authors:** Marc Marty, Akos Dobay, Lars Ebert, Sebastian Winklhofer, Michael Thali, Jakob Heimer, Sabine Franckenberg

**Affiliations:** 1 Department of Forensic Medicine and Imaging, Institute of Forensic Medicine, University of Zurich, 8057 Zurich, Switzerland; marc.alex.marty@me.com (M.M.); akos.dobay@irm.uzh.ch (A.D.); lars.ebert@irm.uzh.ch (L.E.); michael.thali@irm.uzh.ch (M.T.); jakob.heimer@irm.uzh.ch (J.H.); 2 Clinical Neuroscience Center, Department of Neuroradiology, University Hospital Zurich, University of Zurich, 8091 Zurich, Switzerland; sebastian.winklhofer@usz.ch; 3 Institute of Diagnostic and Interventional Radiology, University Hospital Zurich, 8091 Zurich, Switzerland

**Keywords:** MRI, segmentation, strangulation, virtopsy

## Abstract

We evaluate the potential value of magnetic resonance imaging (MRI) in the examination of survivors of manual strangulation. Our hypothesis was that trauma-induced edema of the cervical muscles might lead to a side difference in the muscle volumes, associated with the handedness of the perpetrator. In 50 individuals who survived strangulation, we performed MRI-based segmentation of the cervical muscle volumes. As a control group, the neck MRIs of 10 clinical patients without prior trauma were used. The ratio of the right to left muscle volume was calculated for each muscle group of the control and strangulation groups. Cutoff values for the assumed physiological muscle volume ratios between the right and left sides were identified from our control group. There was no significant difference among the individuals in the pathological muscle volume ratio between right-handed versus both-handed strangulation for the sternocleidomastoid, pretracheal, anterior deep, or trapezoid muscle groups. Only the posterior deep muscle group showed a statistically significant difference in the pathological muscle volume ratio for both-handed strangulations (*p* = 0.011). Measurement of side differences in cervical muscle volume does not allow for a conclusion concerning the probable handedness of the perpetrator.

## 1. Introduction

Medico-legal examination of victims in nonfatal strangulation and the subsequent assessment of the severity of the assault is a challenging task in routine forensic medicine for a number of reasons [1,2,3]. First, there is the discussion of a multifactorial spectrum that could cause potential fatalities in strangulation, which involves four main mechanisms—airway, arterial and/or venous occlusion, and/or autonomic nervous system reflex [3,4]. Second, only a few objective findings are recognized as proof of a life-threatening situation during strangulation:

External findings indicative of strangulation consist of hematoma, swelling, abrasions, and fingernail marks, among others, but these findings do not allow for a conclusive assessment of the severity of the assault [4]. In up to 50% of cases, external findings might be absent entirely [5]. In addition, although symptoms such as unconsciousness, loss of urine or feces, and optical or acoustical sensations are interpreted as a manifestation of cerebral hypoxia (and therefore of a life-threatening assault), these findings are mostly based upon anamnestic information from the victim and are rarely verifiable [3,6]. To date, petechial hemorrhages are the only objectifiable external finding indicative of a sufficient pressure and duration of strangulation that could constitute a life-threatening danger of assault [3].

In addition, medical examinations with computed tomography or laryngoscopy are only performed if there is a clinical suspicion of injuries that might require medical treatment [6]. However, internal objective findings proving a life-threatening situation, such as hyoid bone or laryngeal fractures or carotid dissections, are quite rare [7,8].

Only recently, another imaging modality, magnetic resonance imaging (MRI), has been evaluated for its benefit in the forensic examination of survivors of manual strangulation [2,3,6,8]. It could be shown, in MRI, that typical strangulation signs include subcutaneous and intramuscular hemorrhage, hemorrhage of the lymph nodes and salivary glands, and (rarely) injuries of the larynx and hyoid [2,6,8]. A radiological zone score was also developed in an attempt to help in the appropriate assessment of the severity of the strangulation assault [2]. Nevertheless, despite the recognition of additional valuable documentation of internal findings of the neck from MRI, its use in the assessment of the severity of the assault is currently still very limited [1,2,3,6,8]. Therefore, there is still an urgent need to evaluate further diagnostic possibilities that might help in the adequate medico-legal appraisal of a strangulation assault.

The aim of this study was to further evaluate the potential value of MRI in the examination of survivors of manual strangulation, which might aid in the medico-legal appraisal of such an event. Hypothesizing that trauma-induced edema of the cervical muscles might lead to side differences in the muscle volumes, we wanted to assess whether there is an association between side differences in the cervical muscle volume of the victim and the handedness of the perpetrator.

## 2. Materials and Methods

### 2.1. Study Population

We searched the forensic database of the Institute of Forensic Medicine, University of Zurich, Switzerland, from October 2011 to March 2018 for individuals who survived strangulation (*n* = 633). We included all patients who underwent voluntary (informed consent obtained) noncontrast MRI examination of the head and neck in addition to routine forensic examination (exclusion criteria for MRI: >72 h after the event, <16 years of age, pregnancy, and medical contraindications to MR), resulting in *n* = 114 patients. Of those, we only included individuals for whom the whole neck (from the mandibular angle to the 7th cervical vertebral body) was covered by the MRI examination and whose MR images showed excellent image quality without artifacts. Our final study group consisted of 50 patients (females *n* = 39, males *n* = 11; median age 28.3 years, range 16–57 years).

As a control group, the neck MRIs of 10 clinical patients without prior trauma or other pathology were used (females *n* = 5, males *n* = 5; median age 39.8, range 16–58 years). Informed consent was obtained from all patients.

### 2.2. Magnetic Resonance Imaging

Strangulation cases: MRI was performed at the associated MR center of the University Hospital Zurich, Switzerland, on a 3T MR system (Discovery MR750w, GE Healthcare) according to a standardized, noncontrast study protocol for the neck and head [3].

Control group: MRI, likewise, was performed at the University Hospital Zurich, Switzerland, on a 3T MR system (Achieva 3.0T TX, Philips Healthcare, Best, The Netherlands). The protocol included an axial T2 weighted (Propeller), an axial noncontrast 3D T1 weighted (magnetization-prepared rapid gradient echo, MPRAGE), and an axial diffusion-weighted (DW) (multiplex sensitivity-encoding, MUSE) sequence of the brain, as well as an axial T2 weighted (FLEX, InPhase and WATER), an axial noncontrast fat saturated T1 weighted, and a coronal T2 weighted short-tau inversion recovery (STIR) (Propeller) sequence of the cervical soft tissues.

### 2.3. Segmentation

**Figure 1:**
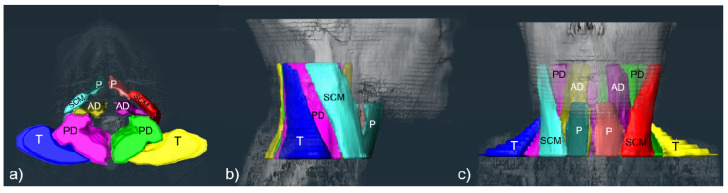
Three-dimensional reconstruction of MRI muscle volume segmentations (case 41) in (**a**) axial, (**b**) right sagittal, and (**c**) coronal views: trapezoid muscle (“T”, right side blue, left side yellow), posterior deep muscles (“PD”, right side pink, left side green), anterior deep muscles (“AD”, right side dark yellow, left side purple), sternocleidomastoid muscle (“SCM”, right side light turquoise, left side red), and pretracheal muscles (“P”, right side dark turquoise, left side rose).

Segmentation was performed on axial T2-weighted images by a master student in medicine under the supervision of a board-certified radiologist and forensic pathologist using the software platform Amira (Amira 6.1.1., Visage Imaging GmbH [European Headquarters, Berlin, Germany]). A combination of manual segmentation and interpolation was used. The segmentation range was defined from the mandibular angle (cranial) to the 7th cervical vertebral body (caudal) for the following muscle groups on each side: sternocleidomastoid muscle, pretracheal muscles (M. sternohyoideus, M. omohyoideus, M. thyrohyoideus, and M. sternothyroideus), anterior deep muscles (M. constrictor pharyngeus inferior, M. constrictor pharyngeus medius, M. constrictor pharyngeus inferior, M. palatopharyngeus, M. longus capitis, M. longus colli, and M. scalenus anterior), posterior deep muscles (M. scalenus medius, M. levator scapulae, M. longissimus cervicis, M. splenius cervicis, M. iliocostalis thoracis, M. semispinalis capitis, M. splenius capitis, M. multifidus, M. semispinalis capitis, M. semispinalis cervicis, M. spinalis cervicis, and M. scalenus posterior), and trapezoid muscle with cranial parts of the M. rhomboideus minor. A horizontal line through the transverse processes of the cervical spine and the scalene gap divided anterior and posterior deep muscles. See Figure 1 for an example.

### 2.4. Data Preparation and Statistics

We included females and males together to increase the statistical significance of our analysis. To adequately reflect potential side differences in the volume of the cervical muscle groups, independent of the individual anatomy, the ratio of the right to left muscle volume was calculated for each muscle group of the control and strangulation groups. We assumed the muscle volume ratios to be normally distributed within our study population. First, we derived a cutoff value for the assumed physiological muscle volume ratios between the left and right sides with the data from our control group. Therefore, the mean plus or minus the standard deviation (SD) for each muscle volume ratio was computed. The SD was used as the cutoff to define the pathological (and thus potentially forensically relevant) cases. In the strangulation mode groups with a sufficiently large case number, we conducted one-way analysis of variance (ANOVA) at a significance level of *p* = 0.05. The statistical analysis was performed using Microsoft^®^ Excel for Mac v. 16, as well as the statistical software R v. 4.0.5 11.

## 3. Results

Table 1 shows the mean and standard deviation of the cervical muscle volume ratio (right to left) of the control group. The interval of the mean ± standard deviation was used to define the physiological range of asymmetry in the muscle volume ratio between the right and left cervical muscle groups.

For the analysis of the strangulation group, only individuals with a ratio outside of the defined physiological range were regarded, and an overview of their data is shown in Table 2.

**Figure 2:**
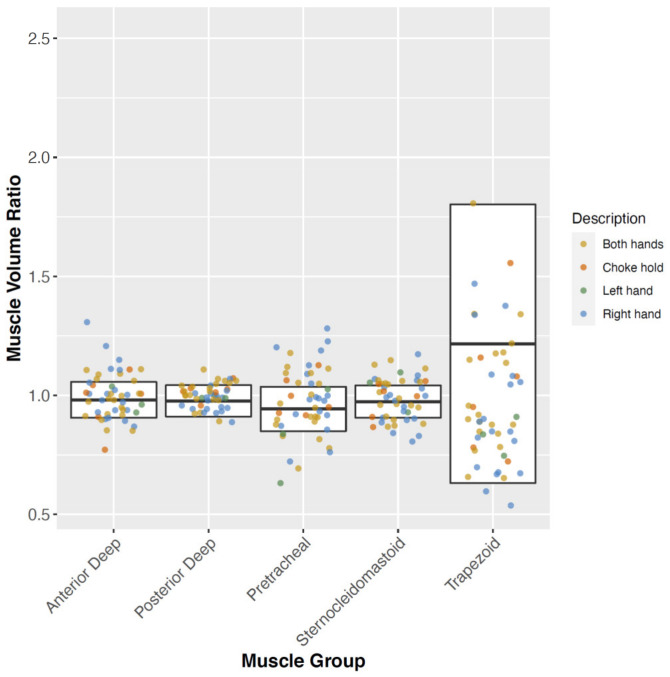
Modified boxplot (mean ± standard deviation instead of the median, 1st and 3rd quantiles) of all cases (control and strangulation), with the different muscle groups on the *x*-axis and the muscle volume ratio on the *y*-axis. The white box shows the physiological variability in asymmetry as defined by the control group (mean ± standard deviation), and the scattered colored points indicate the muscle volume ratios from all strangulation cases (yellow, both hands; orange, choke hold; green, left hand; and blue, right hand).

Figure 2 shows a modified boxplot (mean ± standard deviation instead of the median, 1st and 3rd quantiles) of the muscle volume ratios of both control (white box representing mean ± standard deviation of the control group; whiskers minimum and maximum values) and individual strangulation (scattered colored points) cases.

Only three left-hand and chokehold strangulations were identified each, and consequently, no statistical assessment for these strangulation modes could be performed. Conversely, a sufficient number of cases remained only for right-handed and both-handed strangulation (10 and 13, respectively). There was no significant difference in the pathological muscle volume ratio for right-handed versus both-handed strangulations for the sternocleidomastoid, pretracheal, anterior deep, or trapezoid muscle groups. Only the posterior deep muscle group showed a statistically significant difference in the pathological muscle volume ratio for both-handed strangulations (*p* = 0.011).

## 4. Discussion

Addressing the urgent need to evaluate further diagnostic possibilities that might help in the adequate medico-legal appraisal of a strangulation assault, the aim of this study was to further evaluate the potential value of MRI in the examination process. Hypothesizing that trauma-induced edema of the cervical muscles might lead to side differences in the muscle volumes, we sought to assess whether there is an association between side differences in the cervical muscle volume ratio of the victim and the handedness of the perpetrator.

Only in one muscle group (posterior deep) was a statistically significant difference in the pathological muscle volume ratio found for both hand strangulations (*p* = 0.011). However, we have no comprehensive explanation for this singular result.

For left-handed and choke-hold strangulations, the number of cases was, overall, too low to allow statistical assessment.

Another limitation of this study is that the specified handedness of the perpetrator is based solely on anamnestic information from the victims. In this regard, it must be considered that a high stress level impairs the accuracy of an eyewitness testimony [9]. The anamnestic information obtained from the victim might, therefore, sometimes be incorrect.

One also has to take into consideration that the voxels from MRI data are not isotropic, which might have influenced the accuracy in the segmentation volumes.

In summary, the segmentation of cervical muscle volumes from MRI data of survivors of manual strangulation, thus far, does not add additional value in the examination of the victim and the consecutive legal appraisal of the strangulation offence.

Nevertheless, MRI of the neck remains the best modality for evaluating the soft tissue of the neck [10] and has been shown to often offer additional value in the examination and assessment of strangulation cases [1,2,3,6,8,10]. Therefore, with ongoing and future developments in MRI techniques and software, an increasing number of new forensic relevant findings might be identifiable [11].

## 5. Conclusions

In conclusion, our approach of measurement a side difference in cervical muscle volume does unfortunately not allow for a conclusion concerning the probable handedness of the perpetrator. Further research, especially in MRI of strangulation victims is still necessary. Furthermore, with significantly higher case numbers, training of machine learning and artificial intelligence systems could be performed in the future, which might lead to new insights into the diagnosis of specific strangulation findings and their interpretation in a medico-legal context.

## Figures and Tables

**Table 1:** Ratio of muscle volumes for each muscle group (right to left side) in the control group.

	Ratio_Sterno	Ratio_Pretrach	Radio_Ant_Deep	Ratio_Post_Deep	Ratio_Trap
Mean	0.97	0.94	0.98	0.98	1.22
SD	0.06	0.09	0.07	0.06	0.55
Interval	0.91–1.04	0.85–1.03	0.91–1.05	0.92–1.04	0.66–1.77

**Table 2:** All strangulation cases with the ratio of muscle volume (right to left) for each muscle group. Ratios lower than the physiological range of side asymmetry of the cervical muscle volumes are marked in orange, and ratios higher than the physiological range are marked in green. * The single cases of the “dog leash” and “not specified” strangulation modes were not investigated further.

Case Nr	Sex	Age	Muscle Group Ratio					Strangulation Mode
			Sternocleidomastoid	Pretacheal	Anterior Deep	Posterior Deep	Trapezoid	
1	f	34	0.81	0.87	1.01	0.93	0.85	Right hand
2	f	57	0.96	0.88	0.97	1.00	0.78	Both hands
3	f	34	1.10	0.63	0.96	0.99	0.91	Left hand
4	f	20	0.93	1.03	1.04	1.00	0.75	Left hand
5	f	16	1.11	1.18	1.00	1.11	0.89	Both hands
6	f	29	1.15	0.83	1.01	0.99	1.18	Both hands
7	f	29	1.06	0.98	0.94	1.00	1.09	Right hand
8	m	23	1.06	1.00	1.04	1.03	1.08	Choke hold
9	f	22	1.17	1.20	1.01	0.99	1.38	Right hand
10	f	49	0.91	1.23	0.87	0.99	0.67	Right hand
11	m	25	1.07	0.99	0.90	1.01	0.85	Right hand
12	m	52	0.97	1.07	1.11	0.92	0.79	Dog leash *
13	m	29	1.00	1.13	0.91	1.07	0.78	Choke hold
14	f	33	0.88	0.69	0.92	1.06	1.34	Both hands
15	f	20	0.96	1.05	1.09	1.07	0.92	Both hands
16	m	19	0.91	0.92	1.11	1.03	1.56	Choke hold
17	m	60	0.95	1.28	1.31	0.94	0.90	Right hand
18	f	33	0.84	1.13	1.21	1.04	1.34	Right hand
19	f	26	0.99	0.92	1.00	1.02	0.82	Right hand
20	m	19	0.87	1.06	1.01	1.01	0.95	Choke hold
21	f	24	0.96	1.11	1.09	1.03	0.77	Both hands
22	f	28	0.89	0.92	1.11	0.99	1.08	Right hand
23	f	17	1.05	0.91	0.92	1.00	1.14	Both hands
24	f	25	1.03	1.00	0.93	0.96	0.54	Right hand
25	f	29	0.91	0.95	0.94	1.06	0.66	Both hands
26	m	18	1.05	0.95	1.01	1.02	0.72	Choke hold
27	f	21	0.98	1.09	0.89	1.07	1.47	Right hand
28	f	29	1.01	0.91	1.06	0.93	0.85	Both hands
29	f	26	1.03	1.19	1.05	0.95	0.89	Right hands
30	f	26	0.90	1.09	0.90	1.05	1.34	Both hands
31	f	16	0.90	0.98	0.91	0.95	0.67	Right hand
32	f	27	1.13	0.78	0.98	1.04	1.22	Both hands
33	m	51	1.05	0.97	1.07	1.00	0.88	Both hands
34	f	23	0.95	0.90	0.95	1.04	1.18	Both hands
35	f	19	1.00	0.99	1.15	0.93	0.68	Right hand
36	m	26	1.06	1.12	1.11	0.98	0.84	Both hands
37	f	24	0.83	0.72	1.02	0.98	1.06	Right hand
38	f	30	1.02	0.93	0.77	0.96	1.16	Choke hold
39	f	20	1.00	0.86	0.98	0.94	1.05	Right hand
40	f	29	0.87	1.00	0.85	1.02	0.90	Both hands
41	f	29	1.05	0.84	0.93	0.99	0.84	Left hand
42	f	24	1.08	1.05	0.94	0.89	0.60	Right hand
43	f	18	0.93	0.76	1.11	0.93	0.70	Right hand
44	f	28	0.90	0.93	0.97	1.01	0.81	Right hand
45	f	26	1.06	1.09	1.01	0.98	0.96	Both hands
46	f	51	1.05	1.05	0.91	1.05	1.81	Both hands
47	f	24	1.04	0.89	0.94	0.93	1.04	Not specified *
48	f	23	0.87	0.89	0.85	1.02	0.88	Both hands
49	f	33	0.99	0.82	0.99	0.92	0.65	Both hands
50	m	20	1.06	0.91	1.11	0.89	1.15	Both hands

## Data Availability

Data cannot be made available due to legal restrictions.
